# Dietary Oligosaccharides Attenuate Stress-Induced Disruptions in Immune Reactivity and Microbial B-Vitamin Metabolism

**DOI:** 10.3389/fimmu.2019.01774

**Published:** 2019-07-29

**Authors:** Jacob M. Allen, Robert M. Jaggers, Lindsey M. Solden, Brett R. Loman, Ronald H. Davies, Amy R. Mackos, Christopher A. Ladaika, Brian M. Berg, Maciej Chichlowski, Michael T. Bailey

**Affiliations:** ^1^Center for Microbial Pathogenesis, The Research Institute at Nationwide Children's Hospital, Columbus, OH, United States; ^2^Mead Johnson Pediatric Nutrition Institute, Evansville, IN, United States; ^3^Department of Pediatrics, The Ohio State University College of Medicine, Columbus, OH, United States

**Keywords:** stress, microbiome, metabolome, prebiotics, galactooligosaccharides, B vitamins, *Bifidobacterium*, inflammation

## Abstract

**Background:** Exposure to stressful stimuli dysregulates inflammatory processes and alters the gut microbiota. Prebiotics, including long-chain fermentable fibers and milk oligosaccharides, have the potential to limit inflammation through modulation of the gut microbiota. To determine whether prebiotics attenuate stress-induced inflammation and microbiota perturbations, mice were fed either a control diet or a diet supplemented with galactooligosaccharides, polydextrose and sialyllactose (GOS+PDX+SL) or sialyllactose (SL) for 2 weeks prior to and during a 6-day exposure to a social disruption stressor. Spleens were collected for immunoreactivity assays. Colon contents were examined for stressor- and diet- induced changes in the gut microbiome and metabolome through 16S rRNA gene sequencing, shotgun metagenomic sequencing and UPLC-MS/MS.

**Results:** Stress increased circulating IL-6 and enhanced splenocyte immunoreactivity to an *ex vivo* LPS challenge. Diets containing GOS+PDX+SL or SL alone attenuated these responses. Stress exposure resulted in large changes to the gut metabolome, including robust shifts in amino acids, peptides, nucleotides/nucleosides, tryptophan metabolites, and B vitamins. Multiple B vitamins were inversely associated with IL-6 and were augmented in mice fed either GOS+PDX+SL or SL diets. Stressed mice exhibited distinct microbial communities with lower abundances of *Lactobacillus* spp. and higher abundances of *Bacteroides* spp. Diet supplementation with GOS+PDX+SL, but not SL alone, orthogonally altered the microbiome and enhanced the growth of *Bifidobacterium* spp. Metagenome-assembled genomes (MAGs) from mice fed the GOS+PDX+SL diet unveiled genes in a *Bifidobacterium* MAG for *de novo* B vitamin synthesis. B vitamers directly attenuated the stressor-induced exacerbation of cytokine production in LPS-stimulated splenocytes.

**Conclusions:** Overall, these data indicate that colonic metabolites, including B vitamins, are responsive to psychosocial stress. Dietary prebiotics reestablish colonic B vitamins and limit stress-induced inflammation.

## Introduction

The gastrointestinal microbiota can have profound effects on health through modulation of the host immune, metabolic, and nervous systems (1, 2). Exposure to adverse environmental stimuli, including psychosocial or physical stress, can significantly impact the colonic microbiota by decreasing the abundance of taxa associated with a healthy gut microbiome (e.g., *Lactobacillus*) while increasing the abundance of opportunistic pathogens (3–12). Stressor-induced modifications in the gut microbiota have been associated with parallel changes in central nervous system biology, behavior, and immune system activation (4, 7, 11, 13). Exhibiting direct links between the stress-induced changes in the microbiome and the immune system, it was recently shown that colon contents from stressor-exposed mice transplanted into germfree recipient mice lead to exacerbated inflammation in response to an immune challenge (14). These findings highlight the importance of developing strategies that target the gut microbiota to limit the deleterious effects of stress.

Prebiotics are substrates selectively utilized by gastrointestinal microorganisms that confer a health benefit (15). Commonly used substrates include galactooligosaccharides (GOS), polydextrose (PDX), and sialyllactose (SL) which can enhance the growth and activity of gut microbes (16–19). For example, GOS has been shown to enhance the growth of *Lactobacillus* spp. and *Bifidobacterium* spp. (17, 20). Although PDX alone does not strongly affect growth (20), when GOS+PDX were administered to infants, the increase in *Bifidobacterium* and *Lactobacillus* was similar to infants fed breastmilk (21, 22). Sialyllactose has also been shown to increase *Bifidobacterium* and *Lactobacillus* growth (23), but whether SL has different effects on the colonic microbiome and metabolome compared to GOS+PDX+SL has not been tested. In addition to maintaining selective growth of potentially beneficial bacteria, degradation of these fermentable fibers has been shown to increase the production of short chain fatty acids (SCFAs), which are associated with many beneficial effects in the colon (24). Importantly, prebiotics are also known to affect other less-studied microbial pathways, including amino acid metabolism, vitamin production, and bile acid metabolism (25, 26). For instance, it was recently shown that dietary fiber ameliorated colonic inflammation in rats fed high fat diets through mechanisms largely independent of SCFAs (27). Despite these findings indicating health benefits, the mechanisms and extent to which these prebiotics modulate commensal microbes and their metabolic products to impact host physiology, however, are still not fully understood.

To determine whether prebiotics are efficacious in preventing the deleterious effects of stress on immune system activity, we exposed mice to a social disruption (SDR) stressor that is well-validated and widely used in the behavioral sciences and neurosciences (28–30). We have previously shown that SDR exacerbates inflammatory responses concomitant to disease predisposition and altered behavior (31, 32). As a potential mechanism underlying this response, our laboratory and others have linked stress-induced gut microbiota perturbations to dysfunctional immune responses ultimately contributing to disease predisposition (33, 34). Therefore, therapies that alter the gut microbiota and limit inflammation associated with stress are warranted. Herein, we investigated whether diets containing GOS, PDX, and/or SL prevent the deleterious effects of stress on immune function by promoting anti-inflammatory microbial metabolites.

## Materials and Methods

### Animals

Adult male C57BL/6NCrl strains of *Mus musculus* (Charles Rivers Laboratories, Wilmington, MA) between 6 and 8 weeks of age were used in the study. Animals were given *ad libitum* access to water and the experimental diets for 2 weeks prior to, as well as throughout, stressor exposure. Mice were kept on a 12 h light/dark cycle (lights on at 06:00). All animal experimental procedures were conducted in the Animal Resources Core (ARC) facility with the approval of the Research Institute at Nationwide Children's Hospital Institutional Animal Care and Use Committee (Protocol #AR15-00075). After being received from the vendor, mice were placed on one of three experimental diets. Researchers were blinded to treatment groups throughout the study and sample analyses. The data reported are from three separate experiments. Experiment 1 tested the hypothesis that prebiotic diets could attenuate stressor-induced increases in immunoreactivity as measured by splenic proliferation/viability and cytokine production. Experiment 2 assessed whether the prebiotics also affected the gut microbiome/metabolome. Because these first two experiments identified links between stress, prebiotics, the gut microbiome, and B vitamins, Experiment 3 tested whether stressor-induced immunopotentiation could be directly attenuated by B vitamins.

### Experimental Diets and Experimental Design

Upon arrival, animals were randomly assigned to cages that received AIN-93G control diet supplemented with 15 g/kg galactooligosaccharide (GOS; FrieslandCampina, Zwolle, Netherlands), 15 g/kg polydextrose (PDX; Danisco, Terre Haute, IN), and 2.2 g/kg Lacprodan SL-10 sialyllactose (SL; Arla Foods Ingredients Group P/S, Aarhus, Denmark; designated “GOS+PDX+SL”); or AIN-93G supplemented with 2.2 g/kg SL only (designated “SL”). The diets were formulated to be isocaloric and contained similar carbohydrate, protein and fat levels based on AIN-93G specifications. Each diet contained identical amounts of minerals (AIN-93G-MX-94046, 35 g/kg) and vitamins (AIN-93-VX- 94047, 10 g/kg). Mice were randomly assigned to the non-stress control condition or to exposure to the social disruption stressor. Social disruption occurred on six consecutive days, with fecal samples collected the morning following the 5th day of stressor exposure, and metabolites assessed the morning following the 6th day of stressor exposure.

### Social Disruption (SDR) Stressor

For all experiments, the social disruption (SDR) stressor was used, which entails placing an aggressor mouse (retired breeder C57BL/6NCrl) into the cage with 3 resident mice of the same strain for 2 h between 16:30 and 18:30. During SDR, resident mice were observed for subordinate behaviors such as upright defeat posture, exposed under flank, and fleeing and cowering in the corners of the cage. Aggressors that did not initiate an attack within 5 min were switched with a new aggressor. At the end of each SDR cycle, intruder mice were removed and resident mice left undisturbed until the following day when SDR was repeated with a new aggressor. The health status of the mice was carefully examined throughout the experiment; severely injured mice were removed from the study. Consistent with previous studies using SDR (4), <5% of mice met the early removal criteria. Mice in the control condition were not handled throughout the experiment and were fed either the control or an experimental diet.

### *Ex vivo* Splenocyte Stimulation (MTS) Assay

We assessed the effects of prebiotic diets (Experiment 1) and *ex vivo* B vitamin supplementation (Experiment 3) on stress- and immune- stimulated splenocytes. C57BL/6NCrl were sacrificed the morning following the 6th exposure to the SDR stressor and spleens were removed and processed to create a single cell suspension as previously reported (33, 34). Spleens were gently mashed through a 100 μm cell strainer, suspended in RPMI 1640 medium (Corning, Manassas, VA) supplemented with 10% heat-inactivated fetal bovine serum (Gibco laboratories, Gaithersbrug, MD), and centrifuged at 1,000 rpm for 10 min at 4°C. After decanting the supernatant the pellet was incubated for 2 min at room temperature in Red Blood Cell (RBC) lysis buffer. Following the incubation, the pellet was re-suspended in CTLL-20 RPMI 1640 (1 M HEPES, 200 mM L-glutamine, 7.5% sodium bicarbonate, 100x Antibiotic Antimycotic solution (Sigma Aldrich), β-Mercaptoethanol) and centrifuged. The total numbers of splenocytes were counted using the Countess II FL Automated Cell Counter (Life Technologies). Cell suspensions were diluted to a concentration of 1 x 10^6^ cells/mL of CTLL-20 RPMI. For Experiment 1, mice were fed Control, GOS+PDX+SL, or SL diets for 2 weeks prior to and 1 week during stress (*n* = 9–12/diet/stress group). For Experiment 3, mice were exposed to SDR or remained in home cage but were only fed the control diet (*n* = 6/stress group). In Experiments 1 and 3, splenocytes were stimulated with LPS (*E. coli* O55:B55 (1 μg/mL); Sigma-Aldrich) in replicate 96-well cultures. In Experiment 3, B vitamins (nicotinic acid, nicotinamide, nicotinamide riboside, pyridoxal, pyridoxamine, and pyridoxine) were added at 1,000 nM to separate wells of splenocytes (evenly distributed by individual animal) and stimulated with LPS (1 μg/mL). B vitamer concentrations (1,000 nM) were chosen based off of a previously published report which displayed that 1,000 nM of B3 vitamers added to mammalian cell lines mimicked the physiological response to supplementation *in vivo* (as measured by increase in NAD+ levels) (35). After 18 h at 37°C-in 5% CO_2_, supernatants were collected from half of the wells with a volume of 200 μL per well and cytokine levels (IL-6, IL-1β, TNF-α, IFN-γ) were assessed using the Mesoscale Discovery System V-PLEX Plus Proinflammatory Panel 1 Assay (MSD, Rockville, MD). The remaining wells were incubated for an additional 30 h at 37°C-in 5% CO_2_ at which time the CellTiter 96® AQueous One Solution Cell Proliferation Assay (MTS; Promega, Madison, WI) was administered per manufacturer directions and reduced formazan measured at 490 nm. Serum IL-6 was also assessed via V-Plex Plus Proinflammatory Panel 1 Assay (*n* = 20–22/group), measured across Experiment 1 and 2.

### Metabolomics

Colonic contents of stressor-exposed and non-stressed control mice were collected the morning following the last day of stressor exposure and snap frozen. Frozen samples were shipped to Metabolon (Durham, NC) for processing using the automated MicroLab STAR system (Hamilton Company). Recovery standards were added prior to the extraction process. Proteins were precipitated with methanol under vigorous shaking for 2 min, followed by centrifugation. The resulting extract was divided into five fractions: two for analysis by two separate reverse phase (RP)/UPLC-MS/MS methods with positive ion mode electrospray ionization (ESI), one for analysis by RP/UPLC-MS/MS with negative ion mode ESI, one for analysis by HILIC/UPLC-MS/MS with negative ion mode ESI, and one reserved for backup. Organic solvent was removed with a TurboVap (Zymark) and extracts were stored overnight under nitrogen prior to analysis. Recovery standards, internal standards, and quality control samples (including a pool of human plasma characterized by Metabolon to have known compounds), a pool of aliquots from every sample in this study, pure water, and solvent extract were used. Instrument variability was determined by calculating the median relative standard deviation for the recovery and internal standards. Process variability was determined by calculating the median relative standard deviation for all endogenous metabolites present in 100% of the pooled aliquots from every sample. Experimental samples were randomized across the platform run with QC samples spaced between every third sample injection.

Raw data was extracted, peak-identified and QC processed using Metabolon's hardware and software. Compounds were identified by comparison to a library of over 3,300 purified standards to recurrent unknown entities. Biochemical identifications were based on retention index within a narrow RI window of the proposed identification, accurate mass match to the library (±10 ppm), and the MS/MS forward and reverse scores between the experimental data and authentic standards. Peaks were quantified using area-under-the-curve. Values were normalized to account for variability across run days, and the intensity values were rescaled to set the median equal to 1.

### 16S rRNA Gene Sequencing

Fecal samples were collected by placing the mice into a sterile cage and collecting fecal pellets. DNA was extracted from the fecal pellets (~10 mg) using a QIAamp Fast DNA Stool Mini Kit following manufacturer's instructions, with slight modifications. Briefly, stool was incubated for 45 min at 37°C in lysozyme buffer (22 mg/ml lysozyme, 20 mM TrisHCl, 2 mM EDTA, 1.2% Triton-x, pH 8.0), then bead-beat for 150 s with 0.1 mm zirconia beads. Samples were incubated at 95°C for 5 min with InhibitEX Buffer, then incubated at 70°C for 10 min with Proteinase K and Buffer AL. Following this step, the QIAamp Fast DNA Stool Mini Kit isolation protocol was followed, beginning with the ethanol step. DNA was quantified with the Qubit 2.0 Fluorometer (Life Technologies, Carlsbad, CA) using the dsDNA Broad Range Assay Kit. Samples were standardized to at least 5 ng/μl before being sent to the Molecular and Cellular Imaging Center (MCIC) in Wooster, OH for library preparation. Amplified PCR libraries were sequenced from both ends of the 250 nt region of the V4-V5 16S rRNA hypervariable region using an Illumina MiSeq. DADA2 and Quantitative Insights into Microbial Ecology [QIIME] 2.0 (36) were utilized for amplicon processing, quality control and downstream taxonomic assignment using the ribosomal RNA database SILVA (37).

### Metagenomic Sequencing, Assembly, and Annotation

DNA extracted for 16S rRNA gene analyses (above) was submitted for metagenomic sequencing for one sample in the GOS+PDX+SL-stressed group at the Genomics Shared Research facility at The Ohio State University. Prior to library generation, microbial DNA enrichment was performed using the NEBNext Microbiome DNA Enrichment kit (New England BioLabs, Ipswich, MA) per the manufacturers' protocols. Libraries were generated using the NExtera XT Library System in accordance with the manufacturer's instructions. Genomic DNA was sheared by sonication and fragments were end-repaired. Sequencing adapters were ligated, and library fragments were amplified with five cycles of PCR before solid-phase reversible immobilization size selection, library quantification, and validation. Libraries were sequenced on the Illumina HiSeq 4,000 platform. All raw reads were trimmed from both the 5′ and 3′ ends with Sickle, and then each sample was assembled individually using IDBA-ud using default parameters (38). All scaffolds ≥ 2 kb were binned using MetaBat2 (39). Genes were called using MetaProdigal (40) and annotated using Diamond (41) against the UniRef database (42).

### Statistical Analyses

Prior to statistical analyses, bacterial proportions were assigned to genus level taxonomy and then were normalized by log transformation (13, 43). Normalized bacterial proportions were analyzed using a two factor ANOVA, with stress (Unstressed vs. Stressed) x diet (Control vs. GOS+PDX+SL vs. SL) as between-subjects factors. The Benjamini–Hochberg false discovery rate (FDR) method was implemented to avoid type-1 error.

To determine the extent to which the colonic metabolome differed between stress conditions and diets, Random forest (RF) classification was completed using *R* statistical package. RF was performed on all metabolites using at least 1,000 trees with Matthews Correlation Coefficient *(MCC)* used as an assessment for classification efficacy (44, 45). Next, to understand which metabolites best discriminate between stress and diet status, Boruta feature selection algorithm was implemented (46). Boruta feature selection uses RF and iteratively compares importance of variables with importance of pseudo-random variables. Variables that have significantly worse importance than pseudo-random variables were consecutively dropped, thereby limiting chance of random significance and type-I error (44). Variables “confirmed” by Boruta selection were deemed available for further analysis. Metabolic pathway analysis was performed on Boruta-confirmed features using KEGG Metabolic reference pathway (47).

Splenocyte reactivity was analyzed using a 2-factor ANOVA with stress and diet (Experiment 1)/B vitamin (Experiment 3) as a between subjects factor among LPS treated cells only. For Experiment 3, splenocytes from each animal were evenly distributed among all B vitamin treatments and thus treated as a within factor. Dunnet's *post-hoc* analysis were utilized to compare each treatment (diet or B vitamin) to control group only.

## Results

### Stress-Induced Immune Activation Is Attenuated by Dietary Oligosaccharides

**Figure 1:**
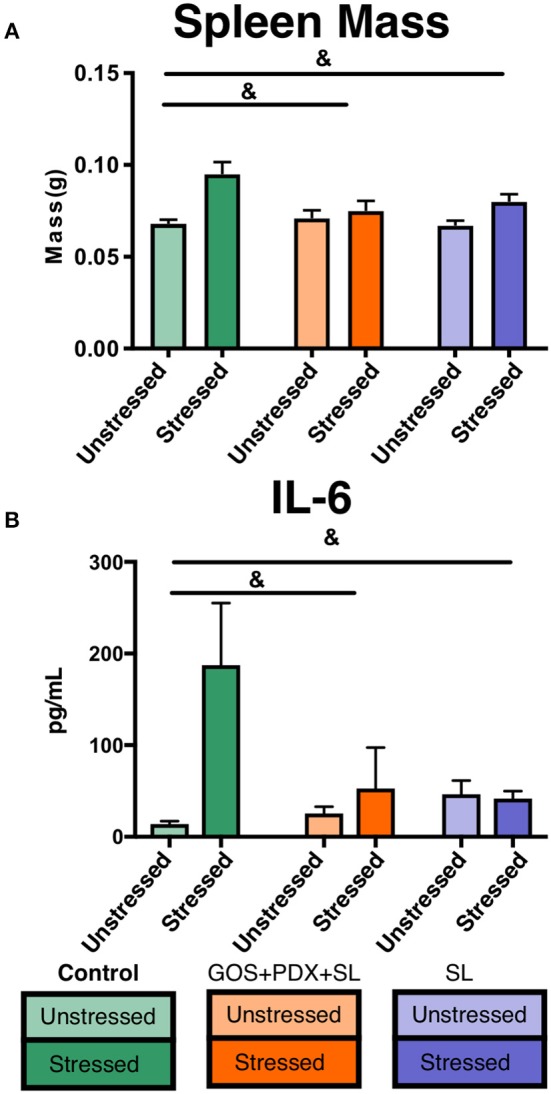
Stressor-induced increases in cytokines and spleen mass are attenuated by GOS+PDX+SL and SL. **(A)** Stressor-induced increases in spleen weight and **(B)** serum IL-6 were attenuated by both diets. ^&^stress x diet interaction, *p* < 0.05 for both GOS+PDX+SL and SL.

To examine the potential for prebiotics to attenuate stress-induced immune activation, we fed mice (*n* = 56) a control diet, a diet supplemented with GOS, PDX, and SL, or a diet supplemented with SL alone for 2 weeks. Half of the mice in each diet treatment (*n* = 9–10/diet group), were exposed to the social disruption (SDR) stressor for 2 h a day for 6 days. Twenty-four hours post SDR, mice were sacrificed for tissue collection (Methods Summary; Supplementary Figure 1). Stressor-exposed mice fed the control diet exhibited elevated serum IL-6 and spleen mass compared to unstressed mice, together indicating stress-induced immune activation, consistent with previous reports (34, 48) (Figures 1A,B, *p* < 0.05). However, stress-induced increases in spleen mass and circulating IL-6 were offset in mice fed GOS+PDX+SL and SL diets (Figures 1A,B, *p* > 0.05, Tukey HSD).

**Figure 2:**
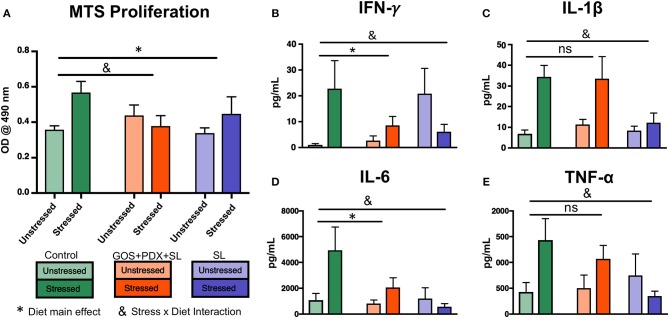
Stressor-induced increases in splenocyte immunoreactivity are attenuated by GOS+PDX+SL and SL. Exposure to stress resulted in **(A)** increased proliferation/viability of *ex vivo* LPS (1 μg/mL)-stimulated splenocytes in control diet fed mice, but not prebiotic fed mice (Tukey HSD *p* < 0.05). **(C–E)** Stressed mice exhibited increased cytokine production from LPS-stimulated splenocytes that was attenuated by one or both prebiotic diets. *Diet Main effect, ^&^Stress x Diet interaction, *p* < 0.05 for all. ns = not significant vs. control-saline group. *n* = 9–10 per diet/stress group. Each experimental diet condition was compared individually to the control diet by Dunnet's *post-hoc* test.

In order to further investigate the role of prebiotics in mediating stress-induced immunoreactivity, spleen cells harvested from control- and prebiotic- fed mice were stimulated *ex vivo* with LPS. Splenocytes from stressor-exposed mice fed the control diet exhibited enhanced proliferation/viability in response to an LPS challenge compared to unstressed mice (*p* < 0.05, Figure 2A). However, this phenomenon was attenuated in mice fed GOS+PDX+SL or SL diets (*p* > 0.05, Figure 2A). Splenocytes from stressor-exposed mice fed the control diet also produced significantly higher levels of IFN-γ, IL-1β, IL-6, and TNF-α upon LPS stimulation (*p* < 0.05; Figures 2B–E), while diets containing GOS+PDX+SL or SL limited the effects of the stressor on LPS-induced cytokine production (Figures 2B–E). Together, these data highlight the potential of dietary oligosaccharides to mitigate the exacerbated inflammatory response due to exposure to stressful stimuli.

### Stress Robustly Alters the Colonic Metabolome

To determine whether the gastrointestinal microbiota underlies the protective effects of dietary oligosaccharides against stress-induced immunoreactivity, we set up a similar experiment to Experiment 1, with mice randomly distributed across stress and diet conditions (*n* = 9–10/stress/diet group; Supplementary Figure 1). At sacrifice, we collected total colon contents for downstream metabolomics (LC/MS/MS), microbial community analysis (16S rRNA gene sequencing), and metagenomics. This allowed for the coordinated measurement of microbial metabolites alongside microbial taxa and genes present in the colon at sacrifice.

**Figure 3:**
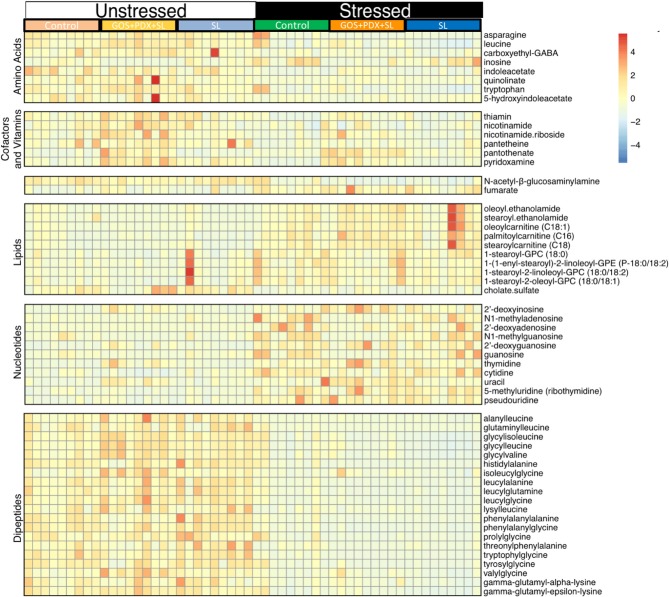
Stress induces robust shifts in the colonic metabolome. A total of 552 metabolites were identified using LC/MS/MS. Samples from the colonic contents were classified based on whether they were from mice exposed to the SDR stressor (stressed) or left in home cage as a control (unstressed). Analysis was performed after collapsing across diet groups. Random forest analysis followed by Boruta selection feature identified 64 colonic metabolites that distinguished between unstressed and stressed mice. The heat map depicts relative intensities of the 64 metabolites arranged by strongest Boruta predictor (from bottom to top; strongest to weakest). *n* = 9–12 mice per stress/diet group.

Collapsed across diet conditions, random forest alongside Boruta feature selection (46) revealed 64 metabolites that significantly differed between stressed and non-stressed mice (Figure 3). Using the Kyoto Encyclopedia of Genes and Genomes (KEGG) database (47), the metabolites delineated into key metabolic groups and pathways that were affected by stress. In particular, amino acids, dipeptides and B vitamins were significantly lower in stressed animals (Figure 3). Meanwhile, lipids and nucleotides were higher in stressed animals (Figure 3). In particular, eight nucleotide and nucleoside derivatives were found to be significantly higher in stressed animals (pseudouridine, thymidine, cytidine, guanosine, deoxyguanosine, methylguansoine, inosine, and deoxyinosine; Figure 3). This was observed in parallel with a significant reduction of three amino acids (asparagine, tryptophan and leucine; Figure 3). Of these amino acids, tryptophan and downstream metabolites were most extensively affected by stress. This included significant reductions in 5-hydroxyindole acetate, quinolinate and indole acetate, together indicating stress-induced disruptions in gastrointestinal tryptophan metabolism. Lastly, random forest analysis revealed a robust effect of stress on the concentration of B vitamins and closely associated metabolites (vitamers). Notably, stress led to lower levels of thiamin (B1), nicotinamide and nicotinamide riboside (B3), pantothiene and pantothenate (B5) and pyridoxamine (B6) (Figure 3).

### Prebiotic Diets Increase Colonic B Vitamin Levels

**Figure 4:**
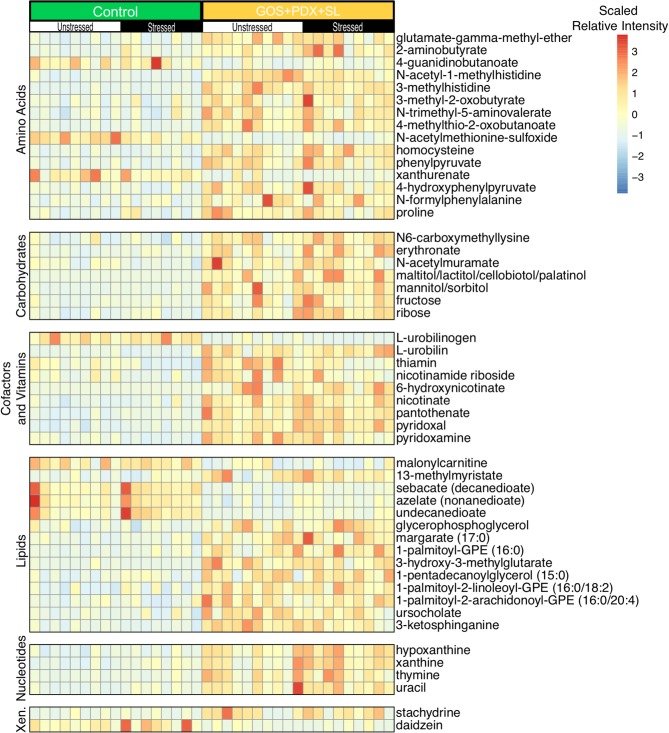
Prebiotic diets with long chain fermentable fiber differentially modulate the colonic metabolome. Random forest analysis followed by Boruta feature selection revealed numerous metabolites that were modulated by GOS+PDX+SL. Analysis was performed after collapsing across stress groups. The heat map depicts relative intensities of metabolites arranged by strongest Boruta predictor [From bottom to top (strongest to weakest)].

After determining the effects of stress on the colonic metabolome, machine learning analysis (RF+Boruta) revealed 51 metabolites that discriminated between mice fed the control diet and mice fed GOS+PDX+SL (Figure 4), while 15 metabolites differentiated mice fed control diet and mice fed SL (Supplementary Figure 2). Random forest with Boruta selection also identified 22 metabolites that differentiated mice fed GOS+PDX+SL from mice fed SL alone (data not shown). Forty-two of the 51 metabolites were higher in the GOS+PDX+SL diet compared to the control diet (Figure 4). Within amino acid metabolism, only 3 amino acids were higher in the control diet (xanthurenate, N-acetylmethionine sulfoxide and 4-guanidinbutanoate). Of particular significance, B vitamins and associated metabolites were increased in the colonic contents from prebiotic fed mice (Figure 4). Specifically, GOS+PDX+SL feeding augmented concentrations of B3 (nicotinamide riboside and nicotinate), B5 (pantothenate) and B6 (pyridoxal and pyridoxamine) vitamins while SL feeding only led to increased levels of B6 (pyridoxal) (RF Boruta confirmed; Figure 4, Supplementary Figures 2, 3A–D).

### Stress and Dietary Oligosaccharides Modify Gut Microbial Composition and Function

**Figure 5:**
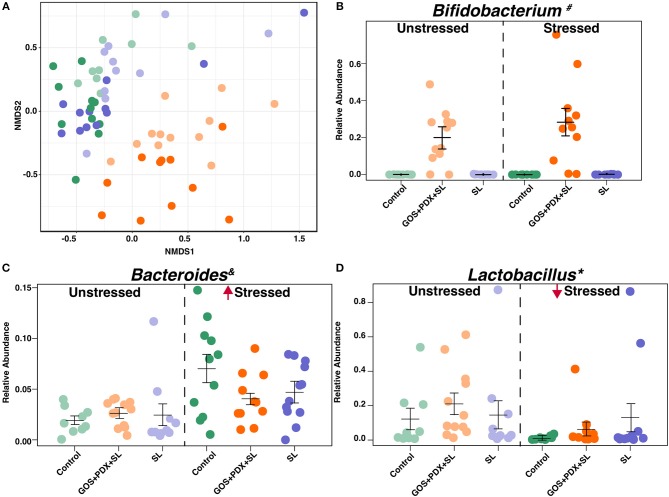
Stress and prebiotic diets orthogonally modulate the composition of the fecal microbiota. **(A)** Non-metric Multidimensional Scaling (NMDS) based off of Bray Curtis dissimilarity metric indicates an effect of stress and diet on colonic microbiome composition (anosim, mrpp *p* < 0.001, respectively). **(B)** GOS+PDX+SL led to higher abundance *Bifidobacterium* ^#^ that was concurrent to lower abundance of *Ruminococcus*^#^ *Oscillospira*^#^ (data not shown), and *Lactococcus*^#^. Stress led to increased levels of **(C)** *Bacteroides* spp. * and lower abundance of **(D)** *Lactobacillus* spp.*. *Stress main effect, ^#^Diet Main effect = FDR p < 0.05; ^&^Stress × Diet Interaction = *p* < 0.05. *n* = 9–12 mice per stress/diet group.

In continuing to identify potential mechanisms underlying diet-induced attenuation in immunoreactivity, we next investigated the gut microbiome, which is a primary mediator of fiber degradation and stress-induced immune responses. Moreover, because select bacteria are capable of B vitamin synthesis, we sought to understand whether the microbiome was a potential mediator through which dietary oligosaccharides mediate B vitamin metabolism. First, we sequenced the V4-V5 hypervariable region of the 16S rRNA gene, isolated and amplified from colon contents of mice exposed to stress and prebiotic diets (Experiment 2, Supplementary Figure 1). Non-metric Multidimensional Scaling (NMDS) based on Bray-Curtis dissimilarity metric revealed microbial communities significantly clustered by stress treatment (mrpp, *p* < 0.001; anosim, *p* < 0.004) and dietary conditions (mrpp, *p* < 0.001; anosim, *p* < 0.001) (Figure 5A). Notably, GOS+PDX+SL diet led to robust effects on the microbiome community composition that were largely orthogonal to the effects of stress (Figure 5, orange), (mrpp, *p* < 0.001). The SL diet alone had a minor effect on overall microbial community composition (Figure 5A).

Several differences in bacterial genera were evident in stressor-exposed mice, including a lower abundance of *Lactobacillus* spp., alongside higher abundances of *Bacteroides* spp. and other taxa including *Butyricimonas* spp. and *Parabacteroides* spp. (FDR *p* < 0.05; Figure 5B). Regardless of stress, animals fed GOS+PDX+SL displayed a robust elevation in *Bifidobacterium* (FDR *p* < 0.05; Figure 5C) concomitantly with lower abundance of *Ruminococcus, Oscillospira*, and *Lactococcus* (FDR *p* < 0.05, data not shown). Because the GOS+PDX+SL diet resulted in higher amounts of B vitamins and prevented stressor-induced increases in IL-6, we probed the relationships between colonic B vitamins and serum IL-6. In doing so, we found that relative levels of nicotinamide riboside, nicotinamide, pantothiene and pyridoxamine in colonic contents were inversely associated with circulating IL-6 (*r* = −0.49, −0.47, −0.41, −0.47, respectively; all *p*'s < 0.05). Moreover, B vitamins were associated with microbiome composition, including a strong relationship between B vitamins (nicotinamide riboside, pyridoxamine, pyridoxal) and *Bifidobacterium* spp. (*r* = 0.58, 0.61, 0.59, respectively; all *p*'s < 0.05).

In light of the robust and specific effects of GOS+PDX+SL diet on *Bifidobacterium* spp, we next sought to understand the genetic potential of this genus to synthesize B vitamers. To accomplish this, we performed metagenomic sequencing (9.69 Gbps) on colonic contents from one animal exposed to stress and provided the GOS+PDX+SL diet, with a particular focus on *Bifidobacterium* assembly. Reads were quality trimmed and assembled into contigs. Contigs were then binned using MetaBat2 (39) and annotated and taxonomically assigned using Diamond (41) against the UniRef database. We recovered one metagenome-assembled genome (MAG) 100% identical by amino acid identity of the ribosomal S3 protein to *Bifidobacterium pseudolongum*, the taxa most significantly upregulated by GOS+PDX+SL diet. This genome was 98.89% complete, with <1% contamination, and assembled into only five scaffolds indicating that this genome is high quality by the Genome Standards Consortium (49) (Supplementary Table 1). Metabolic reconstruction of the *Bifidobacterium* MAG highlighted genes for the *de novo* synthesis of Vitamin B6, including pyridoxal 5′ phosphate synthase. Like many other *Bifidobacterium*, the sequenced *Bifidobacterium* MAG from this study lacks 6-phosphofructokinase, which is one rate-limiting step in glycolysis, but alternatively has fructose-6-phosphoketolase to bypass this step (50) (Supplementary Figure 4). Notably, we detected increased metabolites for this pathway in mice fed the GOS+PDX+SL diet (e.g., erythrose 4-phosphate, sedoheptulose 7-phosphate, and ribulose-5-phosphate), which are precursors to pyridoxal production (Supplementary Figure 4). The GOS+PDX+SL diet also resulted in increases in sugar monomers, glycolysis intermediates, and TCA metabolites, together indicating increased metabolic potential for *de novo* B vitamin synthesis by commensal microbes.

### B Vitamins Directly Attenuate Immunoreactivity in *ex vivo* Stimulated Splenocytes

**Figure 6:**
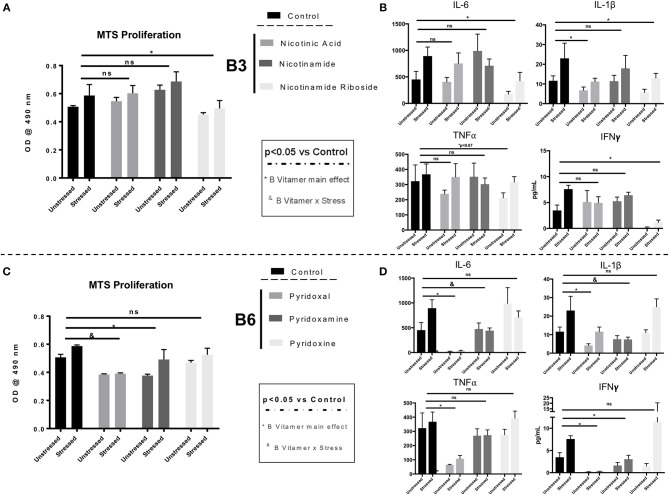
*Ex vivo* proliferation/viability and cytokine production in LPS-stimulated splenocytes from stressor-exposed mice are attenuated by B6 and B3 vitamers. Splenocytes were removed from stressed (*n* = 6) and unstressed (*n* = 6) mice after 6 days of stressor-exposure. Splenocytes were then cultured in saline or 1,000 nM of B vitamers for 48 h concurrent to LPS (1 μg/mL) challenge. **(A,C)** Cell proliferation/viability was determined by MTS Assay after after 4 h of incubation at 37°C. (**B,D)** Supernatant of splenocytes cultured and supplemented for 18 h with B vitamers were assessed for cytokine concentrations (IL-6, IL-1β, TNFα, and IFNγ) using a multiplex ELISA. *B vitamer main effect *p* < 0.05 ^&^ B vitamer × stress *p* < 0.05, ns = not significant. *n* = 6 wells per Stress/ B vitamin group. All B Vitamer supplemented conditions were compared individually to the untreated control condition by Dunnet's *post-hoc* test. Figures depict data from splenocytes stimulated by LPS only.

To further unravel the inhibitory effects of the GOS+PDX+SL and SL diets on stress-induced immunomodulation, we tested whether B vitamers would have direct, inhibitory effects on splenocyte proliferation and immune reactivity to LPS challenge. To test this hypothesis, C57BL/6NCrl mice (stressed *n* = 6; unstressed *n* = 6) fed a normal chow (control) diet were exposed to the SDR stressor for 6 days or left undisturbed as a control as described above (Experiment 3, Supplementary Figure 1). Twenty-four hours after the final stressor exposure, spleens were removed from the mice and single cell suspensions cultured with 1,000 nM of B vitamers for 48 h during concurrent stimulation with LPS (1 μg/mL). We replicated our previous findings showing that stress led to enhanced splenocyte proliferation in response to LPS (stress main effect *p* < 0.05; Figures 6A,C). In a more novel finding, we observed that supplementation of splenocytes with 1,000 nM of the B3 vitamer nicotinamide riboside (B vitamer main effect *p* < 0.05; Figure 6A) as well as the B6 vitamers, pyridoxal and pyridoxamine (Figure 6C) reduced cell proliferation in response to an *ex vivo* LPS challenge (^*^B vitamer main effect *p* < 0.05; Figure 6). In addition, pyridoxal (^#^B vitamer x stress; *p* < 0.05) and nicotinamide riboside (*Trend*, B vitamer × stress; *p* = 0.08) attenuated stress-induced enhancement of splenocyte proliferation in response to the LPS challenge (Figures 6A,C). Next, we analyzed cytokine production from *ex vivo* stimulated splenocytes 18 h after B vitamin supplementation and LPS treatment. Supplementation of splenocytes with 1,000 nM of nicotinamide riboside, pyridoxal and pyridoxamine attenuated LPS-induced IL-6, IL-1β, and IFN-γ production compared to cytokine production from splenocytes not treated with B vitamers (B vitamer main effect *p* < 0.05; Figures 6B,D). Pyridoxamine was also effective at limiting stress-induced enhancement of IL-6 and IL-1β production from splenocytes after LPS stimulation (B vitamer x stress *p* < 0.05; Figure 6D). Pyridoxal was most effective of all B vitamins in suppressing cytokine production from LPS stimulated splenocytes. This was evidenced by a robust attenuation in inflammatory cytokine production (IL-6, TNF-α, IL-1β, IFNγ), in LPS-stimulated splenocytes supplemented with pyridoxal (B vitamer main effect *p* < 0.05; Figure 6D).

## Discussion

There is growing interest in the potential of prebiotics to improve health and well-ness. In this study, diets containing GOS+PDX+SL, or SL alone, attenuated stressor-induced increases in circulating cytokines and potentiated immunoreactivity to an inflammatory challenge. In an effort to understand the mechanism behind these effects, we found that these diets also impacted the structure of the colonic microbiota, evidenced, most notably, by higher levels of *Bifidobacterium* spp. in GOS+PDX+SL-fed mice. This finding corroborates previous data displaying GOS- and PDX-induced *Bifidobacterium* expansion in animals (51, 52) and in human infants (22). Neither the GOS+PDX+SL or SL diet were sufficient to overcome the stress-induced reductions in *Lactobacillus* spp., a phenomenon commonly observed in stressor-exposed mice, rats, nonhuman primates, and humans (53). *Bifidobacterium* and *Lactobacillus* are capable of producing or modifying a wide array of metabolites that are able to influence host immune system reactivity (54). Thus, we focused on the colonic metabolome and its potential to modulate host inflammation during stress and in response to prebiotic diets.

Exposure to stress induced robust shifts in the colonic metabolome. These effects were observed across a broad array of pathways, including amino acid metabolism. This was highlighted by large reductions in tri-peptides, di-peptides, and essential amino acids within the colonic contents of stressor-exposed mice. The mechanisms through which this occurs are not yet understood. Nevertheless, other studies have shown that amino acid degradation by colonic bacteria is enhanced during inflammatory states/conditions, such as Type II diabetes (55), suggesting that systemic inflammation induced by stress may underlie the disruptions in gut amino acid metabolism. Moreover, *Bacteroides* spp. (upregulated by stress in the current study) are also known to have the enzymatic machinery for amino acid degradation (56), and thus may mediate a similar process during stress.

The amino acid tryptophan (Trp) and its downstream metabolites were significantly affected by stress. Trp is an essential amino acid derived from protein in the diet (57) and is primarily metabolized in eukaryotes via the kynurenine pathway. In the current study, stressor exposure led to significant changes in kynurenine pathway metabolites within the colonic lumen, including reduced quinolinate. These effects were observed alongside a decrease in 5-HIAA and serotonin, the latter being important for normal intestinal motility (58) and a primary neurotransmitter involved in mood and cognition that is highly responsive to stress (59). In addition, indole-3-acetetic acid, an aromatic compound produced by the gut microbiota that exerts anti-inflammatory actions through aryl hydrocarbon receptor activation on host immune cell compartments (60, 61), was significantly reduced by stressor exposure. Our findings of stressor-induced modulation in gut tryptophan metabolism are consistent with similar phenomena observed during inflammatory conditions and psychiatric diseases, such as autism and depression (62, 63). Future studies are needed to more thoroughly characterize the effects of stress on tryptophan metabolism within the gut and its potential impacts on health and disease.

In addition to amino acids, stress exposure led to significant reductions in a broad array of B vitamins including thiamin (B1), nicotinamide riboside (B3), and pyridoxamine (B6) within colonic contents. Mammals, including humans, are not capable of synthesizing B vitamins in sufficient amounts to maintain health (64, 65). Many foods contain B vitamins, but colonic microbes have long been recognized as key sources of vitamin B in most mammals (64). Using metagenomic sequencing, we confirmed the presence of genes integral to B vitamin synthesis within the gut microbiota of mice. In a more novel finding, we found the complete pathway for pyridoxal (B6) synthesis within a *Bifidobacterium* metagenome-assembled genome, a bacterial taxa specifically upregulated by GOS+PDX+SL diets. Here we propose that GOS+PDX+SL diet induces preferential growth of *Bifidobacterium* resulting in increased synthesis of B vitamins through the pentose phosphate pathway. This hypothesis is supported with concurrent increases in pentose phosphate metabolites that are primary precursors to *de novo* pyridoxal synthesis through the DXP-independent pathway (65, 66). The SL diet alone (which did not result in increased *Bifidobacterium*) was also sufficient to augment vitamin B6 levels (pyridoxamine and pyridoxal). Pyridoxamine and pyridoxal are essential vitamins, and the vitamin content of the diets was identical. Thus, it is likely that other resident colonic bacteria mediated vitamin B6 metabolism. Indeed, other commensal microbes, including those within the Actinobacteria and Bacteroidetes phylum, are able to produce or modify B6 vitamers (65). Future studies are needed to confirm these hypotheses and to investigate other potential routes of B vitamin synthesis in commensal microbes.

B vitamins play multiple roles in physiological processes throughout the body. Vitamin B6 is a necessary cofactor for hundreds of metabolic transformations in the cell, but primarily those involved with amino acid transformations (67). In addition to affecting cellular metabolism, the B6 vitamins pyridoxal and pyridoxamine can have potent anti-inflammatory effects; for example, pyridoxal has been shown to prevent NF-κB (68) and NLRP3 inflammasome (69) activation in LPS-stimulated macrophages. In the GI tract, vitamin B6 supplementation can reduce colonic inflammation in IL-10–/– knock-out mice and can reduce TNF-α production in HT-29 colonic epithelial cells (70, 71). These previous studies are consistent with our findings that multiple B6 vitamers attenuate cytokine production by LPS-stimulated splenocytes. Vitamin B6 is also recognized as a potent antioxidant and is involved in the production of other essential vitamins, including niacin (66).

In addition to B6 vitamins, B3 vitamins attenuated the effects of stress on LPS-induced cytokine production by splenocytes. Nicotinamide riboside, nicotinate, and nicotinamide are metabolites of vitamin B3 and serve as exogenous sources for NAD+ replenishment. NAD+ regeneration is particularly important during states of high glycolytic flux, oxidative stress, and inflammation, during which endogenous precursors for NAD+ can become depleted (72). Our observation that B3 vitamers were inversely related to serum IL-6 and reduced LPS-induced cytokine production when exogenously administered to splenocytes is consistent with studies displaying protection against inflammatory diseases when nicotinamide riboside supplementation is provided, such as during high-fat diet-induced obesity, Alzheimer's disease, and aging (35, 73).

This study demonstrates that the prebiotics GOS+PDX+SL or SL attenuate the potentiating effects of social stress on immune system reactivity to challenge. This was related to prebiotic-induced increases in colonic B vitamin levels, a phenomenon that most likely occurred through an expansion of gut microbes capable of producing B vitamins, including *Bifidobacterium*. Highlighting a more direct role of prebiotic diet-derived metabolites in mediating immune reactivity, we showed that nicotinamide riboside, pyridoxal and pyridoxamine also directly attenuated the effects of stress on splenocyte proliferation/viability and cytokine production. This has potential importance since splenic leukocytes (such as monocytes) are recruited to sites of inflammation, including the intestine. However, there are limitations to this study. Firstly, we used select combinations of prebiotics (e.g., GOS+PDX+SL) for dietary interventions. While this study provides important information regarding this combinatorial formulation, each dietary component (e.g., PDX) alone was not exclusively investigated for its potential in mediating the gut metabolome and inflammation. Moreover, there are other microbial-derived metabolites, including SCFA, that were likely modified by these prebiotic interventions, but were not a focus of the current study. In turn, future studies focused on the interactions between microbial metabolites (e.g., B vitamers and SCFAs) may prove efficacious for understanding the precise mechanisms underlying the beneficial effects of prebiotics on health. Overall, these observations support the contention that vitamin B metabolites induced by prebiotic feeding limit the exacerbating effects of stress on immune reactivity.

## Ethics Statement

All animals use in this study were approved by the IACUC at Nationwide Children's Hospital.

## Author Contributions

JA performed and planned experiments, collected and analyzed data, and wrote the manuscript. RJ performed experiments, collected and analyzed data, and edited the manuscript. LS collected and analyzed data, and edited the manuscript. BL and RD analyzed data and edited the manuscript. AM collected data and edited the manuscript. CL collected and analyzed data. BB planned experiments and edited the manuscript. MC planned experiments and edited the manuscript. MB planned experiments, analyzed data, and wrote the manuscript.

### Conflict of Interest Statement

The authors declare that the research was conducted in the absence of any commercial or financial relationships that could be construed as a potential conflict of interest.

## Supplementary Material

Supplementary Figure 1 Study design. This study was divided into three main experiments. In experiments 1 and 2, adult male C57BL/6NCr1 strain of *Mus musculus* between 6 and 7 weeks of age were randomly assigned to one of three dietary treatments: the control AIN-93G diet, the GOS+PDX+SL diet consisting of AIN-93G diet with added galactooligosaccharides (GOS), polydextrose (PDX) and siallylactose (SL), or the SL diet consisting of AIN-93G diet and siallylactose (SL) alone. After 2 weeks of diet supplementation, half of the mice in each treatment group were subjected to 2 h of social distruption stressor (SDR) for 6 days. After the 6 days, and a 15 h rest period, mice were sacrificed. In experiment 1, spleens were collected at sacrifice and measured for immunoreactivity with lipopolysaccharide stimulation. 9–10 mice were in each treatment group. In experiment 2 (*n* = 9–12 stress/diet group), total colon contents were collected and submitted for metabolomics and 16S rRNA gene sequencing. Select samples were submitted for metagenomic sequencing. In experiment 3, mice were just fed the control diet for 2 weeks, exposed to SDR for 6 days and then sacrificed. At sacrifice, spleens were collected and stimulated with LPS or LPS supplemented with either B3 or B6 vitamers.

Supplementary Figure 2 Prebiotic diets without long chain fermentable fiber differentially modulate the colonic metabolome. Random forest analysis followed by Boruta selection comparing each experimental diet to control revealed metabolites that were modulated by SL. The heat map depicts relative intensities of metabolites arranged by strongest Boruta predictor [From bottom to top (strongest to weakest)].

Supplementary Figure 3 Colonic B vitamins are broadly and differentially altered by stress and prebiotic diets. Stress led to lower abundance of colonic **(A)** thiamin **(B)** nicotinamide riboside and nicotinamide **(C)** pantethiene and pantothenate and **(D)** pyridoxamine and pyridoxal GOS+PDX+SL led to higher colonic concentration of: **(A)** thiamin **(B)** nicotinamide riboside, nicotinamide, nicotinate **(C)** pantothenate and **(D)** pyridoxamine in colonic contents. Both experimental diets led to higher levels of **(D)** pyridoxal in colonic contents. *n* = 9–12 per diet per group. ^*^Stress main effect, ^#^Diet Main effect, ^&^Stress × Diet interaction, FDR *p* < 0.05 for all. *n* = 9–12 per stress/diet group.

Supplementary Figure 4 Model of B6 vitamin production by *Bifidobacterium pseudolongum* in mice fed the GOS+PDX+SL diet. One *Bifidobacterium* genome (98% complete) contained the complete DXP-independent pathway for *de novo* pyridoxal synthesis. Outside the cell are representative structures for the GOS+PDX+SL diet. Sugars making up these polysaccharides are represented by colored shapes: galactose, orange; glucose, blue; sialylic-acid purple triangle. Metabolites that were upregulated on the GOS+PDX+SL diet are in red text. Genes detected in the *Bifidobacterium* genome are represented by numbered in black boxes and are as follows: [1] beta-galactosidase [2] alpha galactosidase [3] alpha glucosidase [4] lactose permease [5] PTS system for beta-glucosides [6] glycoside hydrolase family 1, bifunctional beta-glucosidase/beta-galactosidase [7] phophoglucokinase [8] glucose 6 phosphate isomerase [9] 6-phosphofructokinase [10] Fructose-6-phosphate phosphoketolase [11] transaldolase [12] transketolase [13] glyceraldehyde-3-phosphate dehydrogenase [14] phosphoglycerate kinase [15] phosphoglycerate mutase [16] enolase [17] pyruvate kinase [18] acetate kinase [19] pyruvate formate lyase [20] alcohol dehydrogenase [21] lactate dehydrogenase [22] pyridoxal 5'phosphate synthase [23] aldose 1-epimerase [24] galactokinase [25] UDP-glucose—hexose-1-phosphate uridylyltransferase [EC:2.7.7.12]. Metabolite abbreviations are as follows: E4P, erythrose 4-phosphate; GAP, glyceraldehyde 3-phosphate; S7P, sedoheptulose 7-phosphate; R5P, ribulose 5-phosphate.

Supplementary Table 1 Bifidobacterium psuedolongum metagenome-assembled genome (MAG). Genome quality and statistics show that this genome is near complete in less than 10 scaffolds and is high quality by the Genome Standards Consortium.

## Abbreviations

TNF: Tumor necrosis factor
IL: Interleukin
IFN: Interferon
GOS: Galactooligosaccharides
SL: Siallylactose
RF: Random Forest
UPLC MS/MS: Ultraperformance liquid chromatography tandem mass spectrometry.
