# Supplementary figures and images for: Dietary Oligosaccharides Attenuate Stress-Induced Disruptions in Immune Reactivity and Microbial B-Vitamin Metabolism
Source: Front Immunol. 2019 Jul 29;10:1774. doi: 10.3389/fimmu.2019.01774 (PMC6681768; doi:10.3389/fimmu.2019.01774)

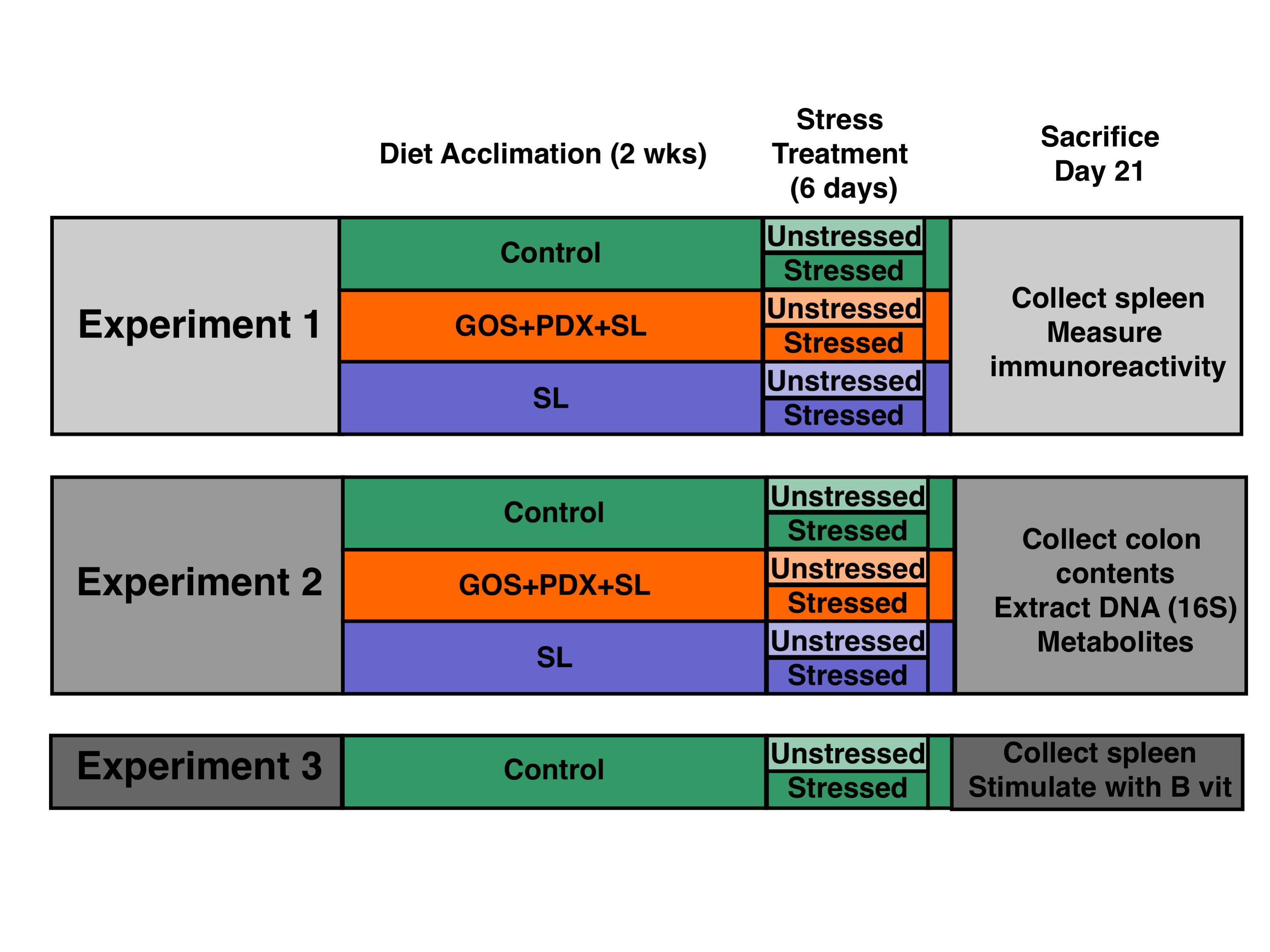

Supplement: Supplementary Figure 1 — Study design. This study was divided into three main experiments. In experiments 1 and 2, adult male C57BL/6NCr1 strain of Mus musculus between 6 and 7 weeks of age were randomly assigned to one of three dietary treatments: the control AIN-93G diet, the GOS+PDX+SL diet consisting of AIN-93G diet with added galactooligosaccharides (GOS), polydextrose (PDX) and siallylactose (SL), or the SL diet consisting of AIN-93G diet and siallylactose (SL) alone. After 2 weeks of diet supplementation, half of the mice in each treatment group were subjected to 2 h of social distruption stressor (SDR) for 6 days. After the 6 days, and a 15 h rest period, mice were sacrificed. In experiment 1, spleens were collected at sacrifice and measured for immunoreactivity with lipopolysaccharide stimulation. 9–10 mice were in each treatment group. In experiment 2 (n = 9–12 stress/diet group), total colon contents were collected and submitted for metabolomics and 16S rRNA gene sequencing. Select samples were submitted for metagenomic sequencing. In experiment 3, mice were just fed the control diet for 2 weeks, exposed to SDR for 6 days and then sacrificed. At sacrifice, spleens were collected and stimulated with LPS or LPS supplemented with either B3 or B6 vitamers. [file Image_1.TIFF]

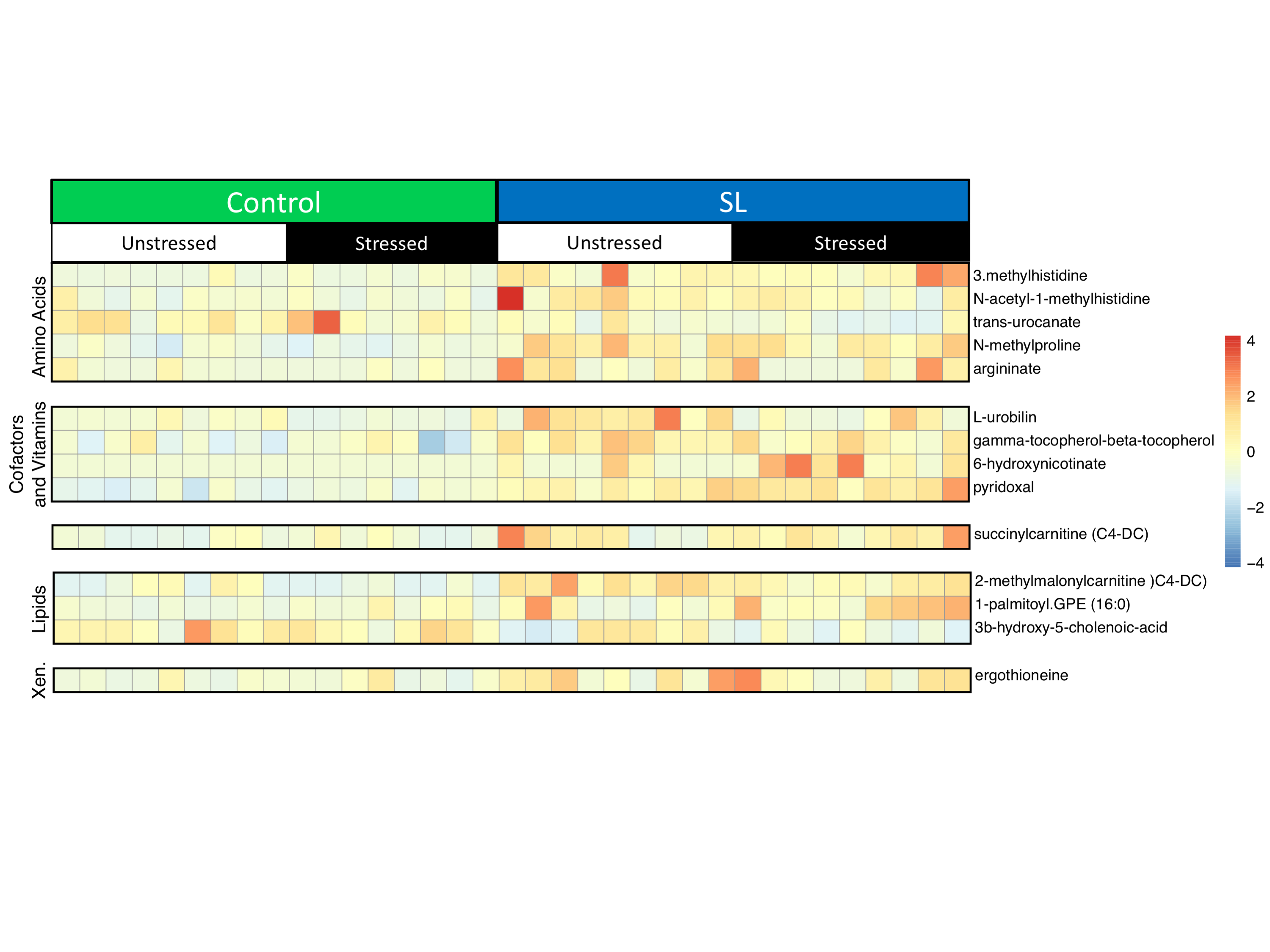

Supplement: Supplementary Figure 2 — Prebiotic diets without long chain fermentable fiber differentially modulate the colonic metabolome. Random forest analysis followed by Boruta selection comparing each experimental diet to control revealed metabolites that were modulated by SL. The heat map depicts relative intensities of metabolites arranged by strongest Boruta predictor [From bottom to top (strongest to weakest)]. [file Image_2.TIFF]

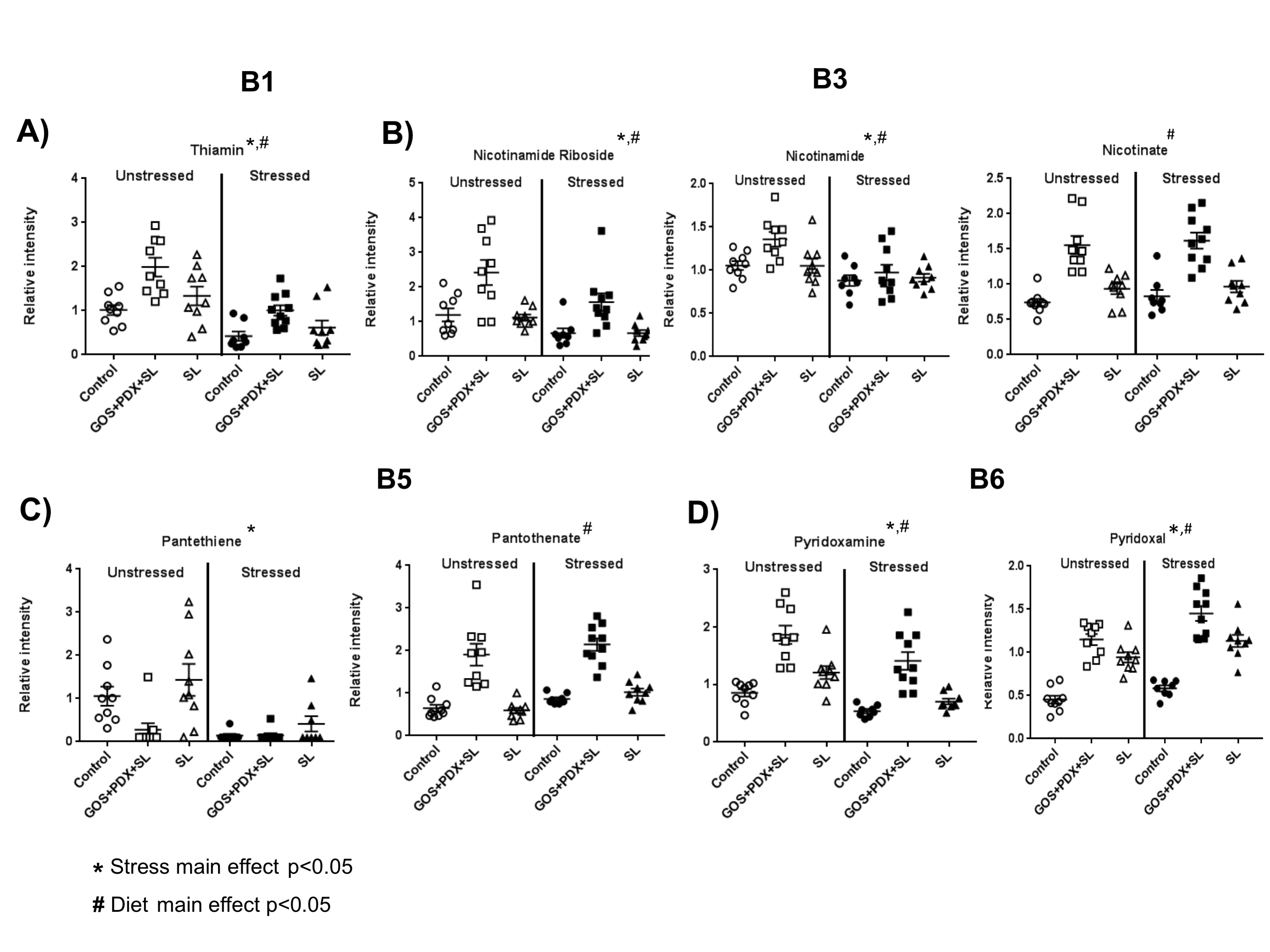

Supplement: Supplementary Figure 3 — Colonic B vitamins are broadly and differentially altered by stress and prebiotic diets. Stress led to lower abundance of colonic (A) thiamin (B) nicotinamide riboside and nicotinamide (C) pantethiene and pantothenate and (D) pyridoxamine and pyridoxal GOS+PDX+SL led to higher colonic concentration of: (A) thiamin (B) nicotinamide riboside, nicotinamide, nicotinate (C) pantothenate and (D) pyridoxamine in colonic contents. Both experimental diets led to higher levels of (D) pyridoxal in colonic contents. n = 9–12 per diet per group. *Stress main effect, #Diet Main effect, &Stress × Diet interaction, FDR p < 0.05 for all. n = 9–12 per stress/diet group. [file Image_3.TIFF]

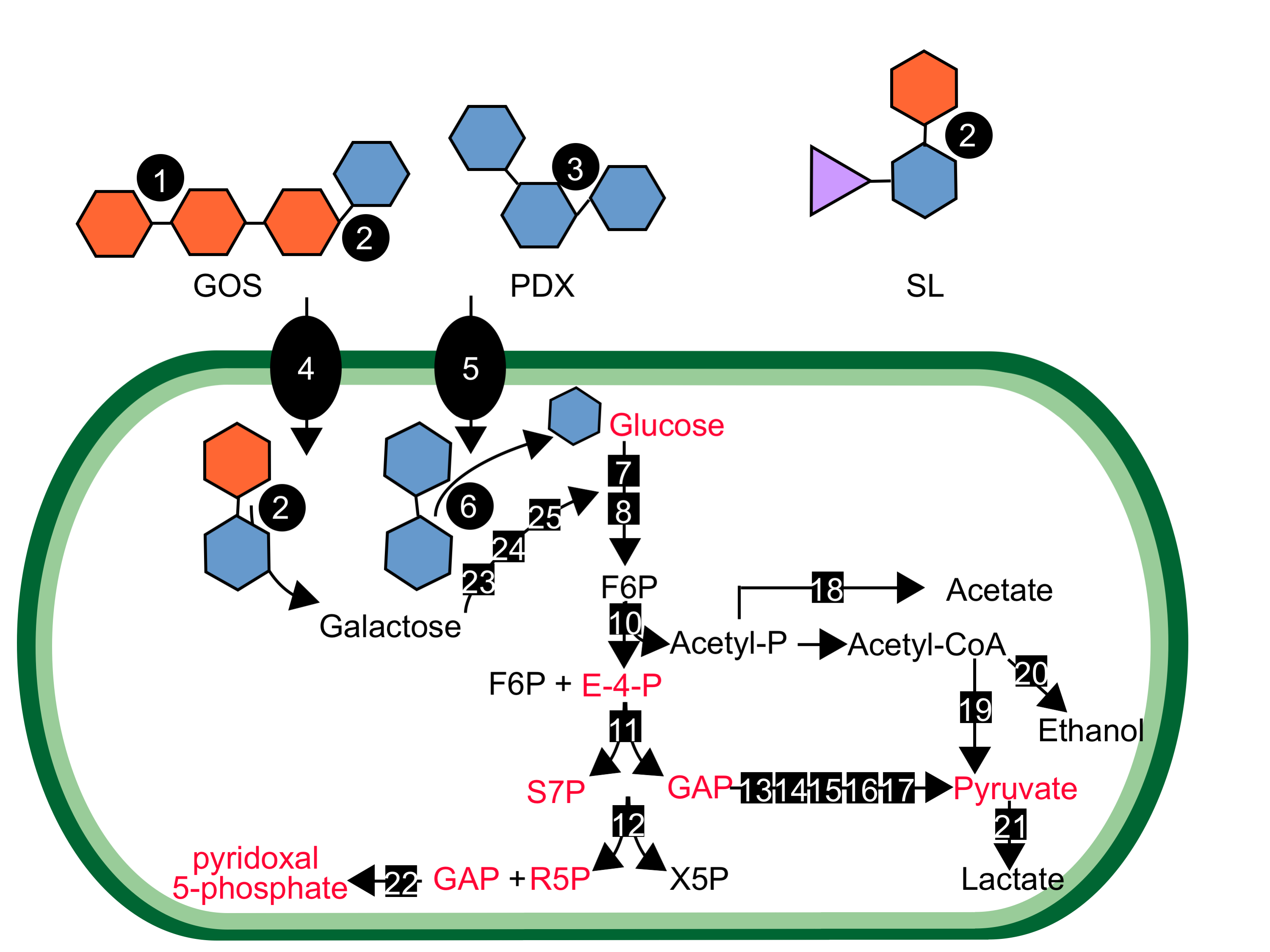

Supplement: Supplementary Figure 4 — Model of B6 vitamin production by Bifidobacterium pseudolongum in mice fed the GOS+PDX+SL diet. One Bifidobacterium genome (98% complete) contained the complete DXP-independent pathway for de novo pyridoxal synthesis. Outside the cell are representative structures for the GOS+PDX+SL diet. Sugars making up these polysaccharides are represented by colored shapes: galactose, orange; glucose, blue; sialylic-acid purple triangle. Metabolites that were upregulated on the GOS+PDX+SL diet are in red text. Genes detected in the Bifidobacterium genome are represented by numbered in black boxes and are as follows: [1] beta-galactosidase [2] alpha galactosidase [3] alpha glucosidase [4] lactose permease [5] PTS system for beta-glucosides [6] glycoside hydrolase family 1, bifunctional beta-glucosidase/beta-galactosidase [7] phophoglucokinase [8] glucose 6 phosphate isomerase [9] 6-phosphofructokinase [10] Fructose-6-phosphate phosphoketolase [11] transaldolase [12] transketolase [13] glyceraldehyde-3-phosphate dehydrogenase [14] phosphoglycerate kinase [15] phosphoglycerate mutase [16] enolase [17] pyruvate kinase [18] acetate kinase [19] pyruvate formate lyase [20] alcohol dehydrogenase [21] lactate dehydrogenase [22] pyridoxal 5'phosphate synthase [23] aldose 1-epimerase [24] galactokinase [25] UDP-glucose—hexose-1-phosphate uridylyltransferase [EC:2.7.7.12]. Metabolite abbreviations are as follows: E4P, erythrose 4-phosphate; GAP, glyceraldehyde 3-phosphate; S7P, sedoheptulose 7-phosphate; R5P, ribulose 5-phosphate. [file Image_4.TIFF]
