# Supplementary material for: Dietary Oligosaccharides Attenuate Stress-Induced Disruptions in Immune Reactivity and Microbial B-Vitamin Metabolism
Source: Front Immunol. 2019 Jul 29;10:1774. doi: 10.3389/fimmu.2019.01774 (PMC6681768; doi:10.3389/fimmu.2019.01774)
Supplement: Supplementary Table 1 — Bifidobacterium psuedolongum metagenome-assembled genome (MAG). Genome quality and statistics show that this genome is near complete in less than 10 scaffolds and is high quality by the Genome Standards Consortium. [file Table_1.docx]

**Supplementary Table 1. *Bifidobacterium psuedolongum* metagenome-assembled genome (MAG).** Genome quality and statistics show that this genome is near complete in less than 10 scaffolds and is high quality by the Genome Standards Consortium.
